# Evaluating the effectiveness of IV iron dosing for anemia management in common clinical practice: results from the Dialysis Outcomes and Practice Patterns Study (DOPPS)

**DOI:** 10.1186/s12882-017-0745-9

**Published:** 2017-11-09

**Authors:** Bruce M. Robinson, Maria Larkina, Brian Bieber, Werner Kleophas, Yun Li, Francesco Locatelli, Keith P. McCullough, Jackie G. Nolen, Friedrich K. Port, Ronald L. Pisoni

**Affiliations:** 1 Arbor Research Collaborative for Health, 340 E. Huron, Suite 300, Ann Arbor, MI 48104 USA; 2 University of Michigan, 1415 Washington Heights, Ann Arbor, MI 48109 USA; 3 Dialysezentrum Karlstrasse, Karlstraße 17-19, 40210 Düsseldorf, Germany; 4 Department of Nephrology, Alessandro Manzoni Hospital, Via dell’Eremo, 9/11, 23900 Lecco, LC Italy; 5 Vifor Pharma, Flughofstrasse 61, 8152 Glattbrugg, Switzerland

**Keywords:** anemia, ferritin, hemodialysis, hemoglobin, IV iron, TSAT

## Abstract

**Background:**

Anemia management protocols in hemodialysis (HD) units differ conspicuously regarding optimal intravenous (IV) iron dosing; consequently, patients receive markedly different cumulative exposures to IV iron and erythropoiesis-stimulating agents (ESAs). Complementary to IV iron safety studies, our goal was to gain insight into optimal IV iron dosing by estimating the effects of IV iron doses on Hgb, TSAT, ferritin, and ESA dose in common clinical practice.

**Methods:**

9,471 HD patients (11 countries, 2009-2011) in the DOPPS, a prospective cohort study, were analyzed. Associations of IV iron dose (3-month average, categorized as 0, <300, ≥300 mg/month) with 3-month change in Hgb, TSAT, ferritin, and ESA dose were evaluated using adjusted GEE models.

**Results:**

***Relative change***: Monotonically positive associations between IV iron dose and Hgb, TSAT, and ferritin change, and inverse associations with ESA dose change, were observed across multiple strata of prior Hgb, TSAT, and ferritin levels. ***Absolute change***: TSAT, ferritin, and ESA dose changes were nearest zero with IV iron <300 mg/month, rather than 0 mg/month or ≥300 mg/month by maintenance or replacement dosing. Findings were robust to numerous sensitivity analyses.

**Conclusions:**

Though residual confounding cannot be ruled out in this observational study, findings suggest that IV iron dosing <300 mg/month, as commonly seen with maintenance dosing of 100-200 mg/month, may be a more effective approach to support Hgb than the higher IV iron doses (300-400 mg/month) often given in many European and North American hemodialysis clinics. Alongside studies supporting the safety of IV iron in 100-200 mg/month dose range, these findings help guide the rational dosing of IV iron in anemia management protocols for everyday hemodialysis practice.

**Electronic supplementary material:**

The online version of this article (10.1186/s12882-017-0745-9) contains supplementary material, which is available to authorized users.

## Background

Anemia due to deficient renal erythropoietin production is nearly universal among hemodialysis (HD) patients. Treatment with erythropoiesis-stimulating agents (ESAs) and intravenous (IV) iron has been the cornerstone of anemia management for over two decades, and it is well established that IV iron is vital to support hemoglobin (Hgb) levels and optimize ESA dosing [1–5].

Despite this, the optimal IV iron management practice to support ESA therapy remains uncertain. First, there may be safety considerations that should limit the amount of IV iron given to patients. While pre-clinical and small clinical studies have indicated that IV iron may lead to oxidative stress, inflammation, risk of infection, and tissue iron overload, large observational studies have reported incongruent associations with clinical outcomes [6–21]. A 2014 KDIGO Controversies Conference on iron management in CKD focused nearly exclusively on the safety of IV iron [22]. Similarly, an ongoing multicenter clinical trial in the UK will compare the safety of a high-dose versus a low-dose IV iron regimen [23].

These questions about the safety of IV iron underlie the imperative to understand the most effective IV iron management strategies, i.e., to give the ‘right amount’ of IV iron to support ESA use and target hemoglobin levels in everyday practice. Data from small clinical trials show that Hgb responsiveness to ESA dosing improves with IV iron dosing that achieves higher levels of iron measures, e.g., transferrin saturation (TSAT) levels of 30%-35% [24–30]. However, ‘real world’ data on the best IV iron dosing strategies to support anemia management are lacking, with a dearth of publications comparing the effectiveness of different IV iron dosing approaches.

Uncertainty regarding optimally effective IV iron management strategies, including IV iron dosing and TSAT and ferritin targets, is reflected in clinical practice recommendations that differ conspicuously: e.g., the KDIGO anemia guideline in 2012 and the ERBP and KDOQI responses to this guideline in 2013 [31–35]. In this context, it is not surprising that previous DOPPS publications have demonstrated that protocols for anemia management and IV iron dosing are widely variable within and across countries [36–39]. As a consequence, patients receive markedly different cumulative exposures to IV iron and ESAs, and ferritin levels are now much higher in the US than in other countries, as are TSAT levels to a lesser extent [40–42].

The primary goal of this study was to help to determine if there is an IV iron management strategy that would optimize the effect of IV iron dosing on Hgb levels and, secondarily, on TSAT, ferritin, and ESA dose in common clinical practice. In the context of unresolved safety questions, we hope to inform optimal IV iron management practices to support Hgb levels while limiting unnecessary or excessive use of ESAs and IV iron.

## Methods

### DOPPS Participants and Data

The Dialysis Outcomes and Practice Patterns Study (DOPPS) is an international prospective cohort study of hemodialysis patients ≥ 18 years of age. Patients are enrolled randomly from national samples of dialysis facilities in each participating country [43, 44]. Demographic data, comorbid conditions, laboratory values, and medications were abstracted from patient records at study enrollment. Laboratory measures and renal medications were updated monthly. Study approval was obtained by a central institutional review board, and as required by national and local ethics committees. Written, informed consent was obtained from all participants.

### Analysis sample

All patients (*n*=15,019) from DOPPS phase 4 (2009-2011) in Europe-A/NZ (Belgium, France, Germany, Italy, Spain, Sweden, and the United Kingdom, plus Australia and New Zealand), and North America (Canada, United States) were eligible for the analysis sample. Patients from Japan were excluded due to much lower levels of IV iron prescriptions. Additional file 1: Figure S1 diagrams a selection of the analysis sample. Patients were required to receive dialysis for at least 4 consecutive months (labeled as months 0 to 3), have TSAT and ferritin values reported at months 0 and 3, have IV iron dosing information for at least 2 of 3 months between the month 0 and 3 lab values, and survive for at least 2 months after month 3, for an analysis sample of 9,471 patients. For patients with multiple suitable 4-month periods, the first period was used. The 3-month interval length was selected because most facilities measure TSAT and ferritin lab values at least quarterly.

### Analytic approach

Monthly IV iron and ESA doses were estimated based on the prescription at the end of each month. Patients not prescribed IV iron or ESA were considered to have a 0 dose for the month. ESA doses were standardized to IV epoetin equivalent units, using a factor of 200 to convert darbepoetin alfa and a factor of 1.15 to convert subcutaneous epoetin [34]. The primary exposure was the average monthly IV iron dose over months 0 to 3 (Additional file 2: Figure S2). The outcomes were changes from month 0 to month 3 in Hgb, TSAT, ferritin, ESA dose, and CRP (denoted with ∆ and subscripts _3-0_ , e.g., ∆Hgb_3-0_).

Primary analyses were modeled separately for 14 different strata: 4 categories of Hgb_0_, 5 categories of TSAT_0_, and 5 categories of ferritin_0_. A generalized estimating equation (GEE) model was used to estimate the expected change in outcomes separately for each stratum condition and reported for each IV iron category (holding all adjustment variables constant). Trends across IV iron categories in each stratum were also tested with *p*-values reported. Each model was adjusted for demographics (age, sex, black race), body mass index, time on dialysis, catheter use, region (North America, Europe-A/NZ), 13 summary comorbid conditions, laboratory values at month 0 (Hgb, ferritin, TSAT, albumin, creatinine, white blood cell count), and ESA dose averaged over months 0 to 3 (unless included in the outcome or strata). Hgb, TSAT, and ferritin were categorized according to common clinical cut points. For the primary analysis, IV iron categories (no IV iron [0 dose], 1 to <300, and ≥300 mg/month) were based on a previous DOPPS publication demonstrating no elevation in mortality at <300 mg/month [6]. Additional analyses dichotomized the ≥300 mg/month IV iron dose category according to replacement dosing (defined as ≥500 mg during any of the 3 months) or maintenance dosing (300-499 mg per month in all 3 months). Multiple sensitivity analyses were performed.

There were few missing values (<2% for the majority of covariates, < 6% for all). Ninety-eight percent of the sample had 3 months of IV iron dosing information, and 2% had 2 months. For missing data, we used the Sequential Regression Multiple Imputation Method by IVEware [45], and analyzed using the MIAnalyze procedure in SAS/STAT® 9.3. Adjustment variables, but not outcome variables, were imputed. Analyses used SAS version 9.3 (SAS institute, Cary, NC).

## Results

### Distribution of IV iron doses

Thirty percent of patients received no IV iron over three months; 40% received (on average) <300 mg/month, and 30% received ≥300 mg/month (Additional file 3: Figure S3). IV iron dose distributions in North America (58% of sample) were similar to Europe-A/NZ (42% of sample). The most common doses were 100 mg/month in the <300 mg/month group and 400 mg/month in the ≥300mg/month group. Seventy-three percent of patients remained in the same IV iron dose category from 1 to 3 months, and 66% with one year of follow-up remained in the same IV iron dose category from 3 to 12 months (Additional file 4: Table S1).

### Patient characteristics by IV iron dose categories (Table 1)

Clinically meaningful differences in age, sex, and most comorbidities were not observed by IV iron dose category. Patients receiving higher doses of IV iron were more likely to receive dialysis via a central venous catheter.

**Table 1:** Baseline characteristics by monthly IV iron dose category

	IV Iron (mg/mo, average over 3 months)^a^			*p* value^b^
	0	<300	≥300	
Study sample				
Patients, N	2800	3749	2922	
Patients, %	30	40	30	
Region, row %				
North America	29	41	30	0.94
Europe and Australia/New Zealand	30	37	32	0.94
Demographics				
Age, years	64.0	64.0	63.5	0.06
BMI, kg/m^2^	27.0	27.5	27.9	<.01
Male, %	57	58	58	0.47
Black, within US only, %	33	31	29	<.01
Duration on dialysis, years	4.7	4.1	3.0	<.01
Catheter, %	25	29	39	<.01
Comorbidities, %:				
Diabetes	47	51	54	<.01
Hypertension	85	83	84	0.53
Coronary Heart Disease	37	36	35	0.16
Cerebrovascular Disease	13	14	14	0.77
Congestive Heart Failure	30	30	28	0.80
Peripheral Vascular Disease	26	25	25	0.22
Other Cardiovascular	25	25	26	0.59
Cancer (non-skin)	13	11	13	0.87
GI Bleed (within 12 months)	4	4	5	0.23
Lung Disease	13	13	13	0.77
Neurologic Disease	10	9	9	0.19
Psychiatric Disorder	17	16	15	0.20
Recurrent Cellulitis or Gangrene	9	10	9	0.89
Laboratory Values (at month 0)				
Albumin, g/dL	3.79	3.77	3.68	<.01
Creatinine, mg/dL	8.3	8.0	7.5	<.01
White Blood Cells, x 10^3^/uL	7.0	7.0	7.2	<.01
TSAT, %	31.5	29.0	23.8	<.01
Ferritin, ng/mL	687	538	441	<.01
Hemoglobin, g/dL	11.6	11.5	11.2	<.01
CRP, mg/L^c^	12.7	11.9	15.8	0.35
Medication Doses (3-month average)				
IV Iron, mg/month	0	170	523	
ESA, IV epoetin equivalent units/week	10759	11426	16101	<.01

^a^Values in table are sample mean unless noted otherwise

^b^ *p* value for trend within row (across three iron dose categories), adjusted for region and accounting for facility clustering, using logistic model for dichotomous and linear mixed model for continuous measures

^c^Restricted to facilities measuring CRP in at least 50% of their patients (all North American facilities excluded)

### Change in Hgb (Fig. 1)

**Relative change**: A positive trend (*p*<0.05) between the three average monthly IV iron dose categories and ∆Hgb_3-0_ was observed in all but one of the 14 total Hgb_0_, TSAT_0_, and ferritin_0_ strata. For the 14 strata, the median (range) difference in ∆Hgb_3-0_ was 0.18 (0.10 to 0.41) g/dL for IV iron <300 mg/month compared to no IV iron, and 0.13 (-0.07 to 0.40) g/dL for IV iron ≥300 mg/month compared to <300 mg/month. **Absolute change**: Across Hgb_0_ strata, the absolute ∆Hgb_3-0_ showed regression to the mean (generally consistent with treatment to target Hgb); namely Hgb increased for patients with Hgb_***0***_ <11 g/dL in all IV iron dose categories, and decreased for patients with Hgb_***0***_ ≥12 g/dL in all IV iron dose categories. By contrast, in all but two TSAT_***0***_ and ferritin_***0***_ strata the absolute ∆Hgb_3-0_ was negative when the mean monthly IV iron dose was 0. Across TSAT_***0***_ and ferritin_***0***_ strata, the absolute ∆Hgb_3-0_ was near zero or slightly positive for IV iron <300 mg/month and ≥300 mg/month.

**Fig. 1:**
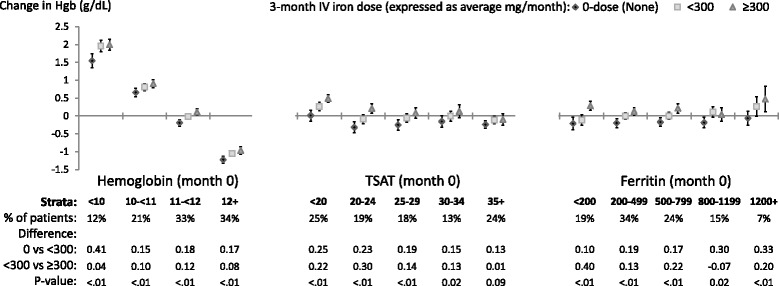
Adjusted change in **Hemoglobin**, from before to after IV iron dosing. Each stratum represents a separate model (14 total per figure) adjusted for age, sex, black race, time on dialysis, catheter use, BMI, region (Europe-ANZ, North America), 13 comorbid conditions, and the following measures at month 0: Hgb, white blood cell count; serum albumin, creatinine, ferritin, TSAT; and 3-month ESA dose (unless included in the outcome or strata). The vertical bars indicate 95% confidence intervals. Total sample size was 9,471

### Change in ESA dose (Fig. 2)

**Relative change**: An inverse relationship (*p*<0.05) between the three average monthly IV iron dose categories and ∆ESA dose_3-0_ was observed in all but three of 14 total Hgb_0_, TSAT_0_, and ferritin_0_ strata. **Absolute change**: The estimates for absolute ∆ESA dose_3-0_ were positive for IV iron 0 mg/month in 13 of the 14 total Hgb_***0,***_ TSAT_***0***_, and ferritin_***0***_ strata, and were negative for IV iron ≥300 mg/month in all 14 Hgb_***0,***_ TSAT_***0***_, and ferritin_***0***_ strata. Estimates for absolute ∆ESA dose_3-0_ were generally nearer to zero with IV iron <300 mg/month than with either 0 or ≥300 mg/month. The differences in absolute ∆ESA dose_3-0_ across the three IV iron dose groups were greater in the low compared to high Hgb_0_ and TSAT_0_ strata. No such pattern was evident across ferritin_0_ strata.

**Fig. 2:**
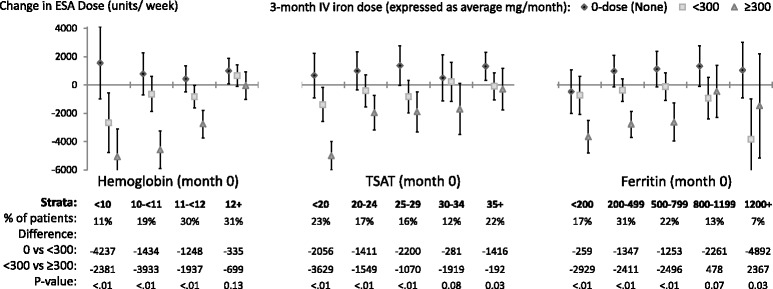
Adjusted change in **ESA dose**, from before to after IV iron dosing. Each stratum represents a separate model (14 total per figure) adjusted for age, sex, black race, time on dialysis, catheter use, BMI, region (Europe-ANZ, North America), 13 comorbid conditions, and the following measures at month 0: Hgb, white blood cell count; serum albumin, creatinine, ferritin, TSAT; and 3-month ESA dose (unless included in the outcome or strata). The vertical bars indicate 95% confidence intervals. Total sample size was 9,471, with 10% missing outcome variable hence “% of patients” adds up to 90%

### Changes in TSAT (Fig. 3) and ferritin (Fig. 4)

**Relative change**: Positive trends (*p*<0.05) between the three average monthly IV iron dose categories and both ∆TSAT_3-0_ and ∆ferritin _3-0_ were observed in all 14 total Hgb_0_, TSAT_0_, and ferritin_0_ strata. These associations were directionally similar to those for change in Hgb (Fig. 1).

**Fig. 3:**
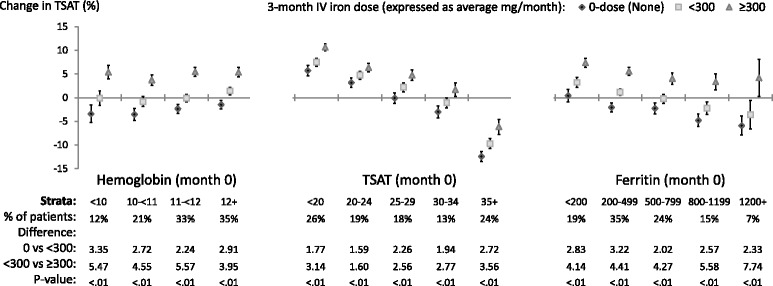
Adjusted change in **TSAT**, from before to after IV iron dosing. Each stratum represents a separate model (14 total per figure) adjusted for age, sex, black race, time on dialysis, catheter use, BMI, region (Europe-ANZ, North America), 13 comorbid conditions, and the following measures at month 0: Hgb, white blood cell count; serum albumin, creatinine, ferritin, TSAT; and 3-month ESA dose (unless included in the outcome or strata). The vertical bars indicate 95% confidence intervals. Total sample size was 9,471

**Fig. 4:**
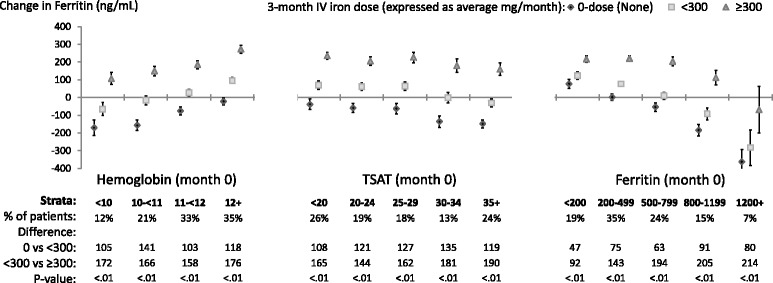
Adjusted change in **Ferritin**, from before to after IV iron dosing. Each stratum represents a separate model (14 total per figure) adjusted for age, sex, black race, time on dialysis, catheter use, BMI, region (Europe-ANZ, North America), 13 comorbid conditions, and the following measures at month 0: Hgb, white blood cell count; serum albumin, creatinine, ferritin, TSAT; and 3-month ESA dose (unless included in the outcome or strata). The vertical bars indicate 95% confidence intervals. Total sample size was 9,471

### Maintenance versus replacement dosing patterns in the ≥300 mg/month category (Additional file 5: Figure S4)

Among patients in the ≥300 mg/month category, the associations of IV iron dosing with ∆Hgb_3-0_ were comparable across Hgb_0_ strata for the replacement versus maintenance IV iron dosing subgroups. In general, maintenance dosing had a weaker (less positive) effect on ∆TSAT_3-0_ and a stronger (more positive) effect on ∆ferritin _3-0_ than replacement dosing.

### Sensitivity analyses

The associations of IV iron dose on outcomes were evaluated in additional subgroups such as strata of Hgb0-by-TSAT0, Hgb0-by-ESA dose, TSAT0-by-ESA dose (Additional file 6: Figure S5), iron dose over 3 months prior to month 0, and geographic region. For all categories with adequate sample size, the observed associations with IV iron dose were consistent with Figs. 1, 3, and 4. Exclusion of incident patients (<90 days on dialysis) had no material impact on results. Consideration of other functional forms for IV iron dose, including as a categorical variable with different cut points or a continuous variable, yielded results consistent with the primary analysis. Restricting the IV iron dose period to 1 month demonstrated that the 1-month effect on Hgb, TSAT, and ferritin was directionally unchanged from, though quantitatively lower than, the 3-month effect (Additional file 7: Figure S6). Corroborative results were observed when lagging the outcome by one month (i.e., to month 4), when evaluating outcomes over 1 month of IV iron dosing, rather than 3 months, and when stratifying 1 or 3 month IV iron dose by prior 3-month iron dose, in effect providing estimates of the effect of a 4- to 6-month IV iron dose.

### Association with CRP levels (Additional file 8: Figure S7)

Strata of CRP_0_ (<3, 3-<10, and 10+ mg/dL), an inflammatory measure obtained commonly in European dialysis units, were evaluated. No directional associations were seen between average monthly IV iron dose and 3-month change in C-reactive protein, in contrast with the clear patterns of ‘dose response’ observed for the anemia-related outcomes.

## Discussion

Our findings, from a multinational observational study of practice patterns in national samples of hemodialysis centers, provide support for the effectiveness of IV iron dosing to raise or sustain Hgb across a wide range of starting Hgb, TSAT, and ferritin values in everyday practice. Specifically, the results support the possibility that IV iron dosing of less than 300 mg/month, as commonly used for maintenance IV iron dosing at 50 mg per week or 50-100 mg every other week (the dominant dose range for this category was 100-200 mg/month) may be the most effective strategy to support Hgb while keeping TSAT and ferritin levels stable and limiting ESA dose. Higher IV iron doses were associated with lower doses of ESA, but at a price of a rise in ferritin (especially with maintenance dosing) and/or TSAT (especially with replacement dosing). Along with studies supporting the safety of IV iron in the 100-200 mg/month dose range, these results provide greater clarity to help guide the rational use of IV iron in common clinical practice across a diverse range of dialysis centers and patients, and across diverse countries.

A motivation for our analysis was the paucity of data providing practical guidance for the comparative effectiveness of different IV iron doses to optimize Hgb outcomes and ESA responsiveness in dialysis patients, which could help providers to limit administration of higher doses than necessary given uncertainty about clinical risk. Among HD patients with absolute or functional iron deficiency, the effectiveness of IV iron to raise Hgb levels is established [2, 31–33]. For most HD patients, who have neither absolute nor functional iron deficiency, small clinical studies consistently have shown that optimizing IV iron dosing supports Hgb levels while decreasing ESA dose requirements [22–30]. However, no interventional trials or adequately controlled large observational studies have sufficiently evaluated the effectiveness of different IV iron dosing strategies to support Hgb responsiveness to ESA dosing in broader dialysis practice. Absent clear evidence or consensus guidelines, anemia management protocols are highly diverse, and patients receive markedly different cumulative exposures to IV iron and ESAs [31–38].

To help address this evidence gap, our analysis corroborates the positive dose response (**relative change**) between IV iron and Hgb, TSAT, and ferritin levels, well known from smaller studies, in a large database reflective of routine clinical care in diverse settings internationally. This work also provides estimates of the magnitude of the effect of IV iron on 3-month change in Hgb in routine clinical practice, demonstrating a median difference of 0.18 g/dL for IV iron doses of <300 mg/month versus 0, and of 0.13 g/dL for IV iron doses of ≥300 versus <300 mg/month, varying modestly across Hgb_0_, TSAT_0_, or ferritin_0_ strata.

Based on our Medical Director Survey data (not shown) specifying target ranges for Hgb, TSAT, and ferritin levels, the majority of patients in this analysis had values of these measures that were at or near target range. For such patients, an important goal of anemia management is to maintain Hgb at stable levels (i.e., **absolute change** of zero), while optimizing ESA dosing. Deviations have been referred to as Hgb variability, cycling, or excursions, and have been associated with adverse clinical outcomes [46–49]. Furthermore, maintaining stable TSAT and ferritin levels in the target range plausibly has beneficial clinical and cost implications. In our analyses, we note that Hgb, TSAT, and ferritin levels were most often unchanged (absolute change = 0) from before to after IV iron dosing of <300 mg/month. This finding held across broad ranges of prior Hgb, TSAT, and ferritin levels, inclusive of common targets such as Hgb 10-12 g/dL, TSAT 20%-34%, and ferritin 200-799 ng/mL. The <300 mg/month IV iron dosing category includes some common maintenance IV iron dosing regimens, e.g., 50 mg/week or 50-100 mg every other week (Additional file 3: Figure S3). We therefore extrapolate that this IV iron dosing strategy may be the best means to maintain stable Hgb, TSAT, and ferritin levels in many patients.

Among patients receiving IV iron at ≥300 mg/month, our analysis estimated the effects of high dose maintenance (e.g., 100 mg weekly) versus replacement (e.g., 500mg or 1000 mg bolus) dosing (Additional file 5: Figure S4). While the observed effects on Hgb levels were comparable, both strategies were associated with increases in TSAT and ferritin levels even among patients with high prior TSAT (up to 35%) and ferritin (up to 1200 ng/mL) levels. These observed effects may indicate administration of unnecessarily high cumulative IV iron doses; for example, maintenance dosing (which has lesser short-term, but perhaps greater long-term, effect on raising ferritin levels than replacement dosing) was associated with an increase in ferritin of an additional 63 ng/mL for patients with ferritin levels already at 500-799 ng/mL. These findings provide further support for use in common practice of IV iron dosing at <300 mg/month, compared to ≥300 mg/month whether by maintenance or replacement dosing.

Relative decreases in 3-month ESA dose were consistently seen for IV iron dose <300 mg/month compared to no IV iron, especially at low Hgb_0_ and TSAT_0_ levels. While IV iron ≥300 mg/month was associated with even larger relative decreases in 3-month ESA dose, the absolute change in ESA dose was generally closest to 0 following IV iron dosing of <300 mg/month. This finding supports the possibility that IV iron dosing at <300 mg/month may, on average, limit the need for ESA dosing adjustments, a desirable goal in everyday dialysis practice. Especially for patients with low Hgb_0_ (<11 g/dL) or low TSAT_0_ (<20%) levels, the large increase in Hgb level and large decline in ESA dose following IV iron dosing emphasize the importance of sufficient IV iron dosing in such patients to limit unnecessarily high ESA dose.

Our analysis evaluated effectiveness rather than safety outcomes, but our conclusion that IV iron dosing at 100-200 mg/month (the most common doses in <300mg/month group) may be optimally effective to support Hgb levels is notable when placed in the context of existing literature because, to our knowledge, studies have found elevation in clinical risk only at higher (e.g., ≥300 mg/month) IV iron doses [6–14].

Regarding interpretation of our findings, several caveats merit mention. First, though we evaluated the effect of IV iron dose over up to 6 months, analyses are needed to confirm our findings over longer follow-up intervals. Second, the analysis provides information on average, but doesn’t provide insight into the most effective IV iron dosing strategies for all situations in clinical practice, such as patients with Hgb levels substantially above or below target or patients who are hyporesponsive to ESA therapy. Third, this was a study of prevalent HD patients, most of whom are not naïve to anemia treatment; however, prior treatment history cannot be fully represented in the analysis. Fourth, all observational studies are subject to biased interpretation due to confounding. However, the associations seen are directionally consistent with causal rather than confounded interpretations. Confounding would tend to bias the effect of IV iron on Hgb to the null, since patients receiving higher doses of IV iron are more likely to be ESA resistant than patients receiving lower dose or no IV iron. Fifth, we were unable to report estimated effects of different IV iron formulations because, within a geographic region, there was often only one product in predominant use [38, 40].

## Conclusion

Though IV iron use in dialysis units is nearly ubiquitous, the optimal way to dose and manage iron parameters to treat anemia is controversial and deserves input from analyses of actual clinical practice. Our observational findings from the international DOPPS provide support for the effectiveness of IV iron dosing to raise or sustain Hgb levels across a wide range of Hgb, TSAT, and ferritin values. Further, the findings indicate that IV iron dosing at <300 (typically 100-200) mg/month, as used for some maintenance IV iron dosing protocols, may be a more suitable strategy for average patients to support Hgb, while keeping TSAT and ferritin levels stable and reducing or limiting ESA doses, than the higher doses, by either maintenance or replacement dosing, that are common in many European and North American dialysis clinics. Along with studies that support the safety of IV iron in the same 100-200 mg/month dose range, these findings can help to inform clinical practice guidelines and anemia management protocols in everyday hemodialysis practice.

## Additional files

Additional file 1: Figure S1. Flow Chart for Selection of Analysis Sample. (PPTX 540 kb)

Additional file 2: Figure S2. Schematic of timing of IV iron dose, ESA dose, and laboratory measures in analytic models. (PPTX 529 kb)

Additional file 3: Figure S3. Distribution of 3-month IV iron dose. (PPTX 546 kb)

Additional file 4: Table S1. Joint distributions of 3-month and 12-month IV iron doses (% of patients). (DOCX 34 kb)

Additional file 5: Figure S4. Adjusted change in hemoglobin, TSAT, or ferritin from before to after IV iron dosing, dividing ≥ 300 mg/month into maintenance and replacement dosing categories. (PPTX 89 kb)

Additional file 6: Figure S5. Adjusted change in Hemoglobin, from before to after IV iron dosing stratified by ESA Dose and TSAT. (PPTX 117 kb)

Additional file 7: Figure S6. Adjusted change in 1-month Hemoglobin, TSAT, or Ferritin from before to after IV iron dosing. (PPTX 88 kb)

Additional file 8: Figure S7. Adjusted change in CRP, from before to after IV iron dosing. (PPTX 56 kb)

## Abbreviations

DOPPS: Dialysis Outcomes and Practice Patterns Study
ESA: erythropoiesis-stimulating agents
GEE: generalized estimating equation
HD: hemodialysis
Hgb: hemoglobin
IV: intravenous
TSAT: transferrin saturation
