# Supplementary material for: Evaluating the effectiveness of IV iron dosing for anemia management in common clinical practice: results from the Dialysis Outcomes and Practice Patterns Study (DOPPS)
Source: BMC Nephrol. 2017 Nov 9;18:330. doi: 10.1186/s12882-017-0745-9 (PMC5679150; doi:10.1186/s12882-017-0745-9)
Supplement: Supplementary file 1 — Flow Chart for Selection of Analysis Sample. (PPTX 540 kb) [file 12882_2017_745_MOESM1_ESM.pptx]

## Slide 1
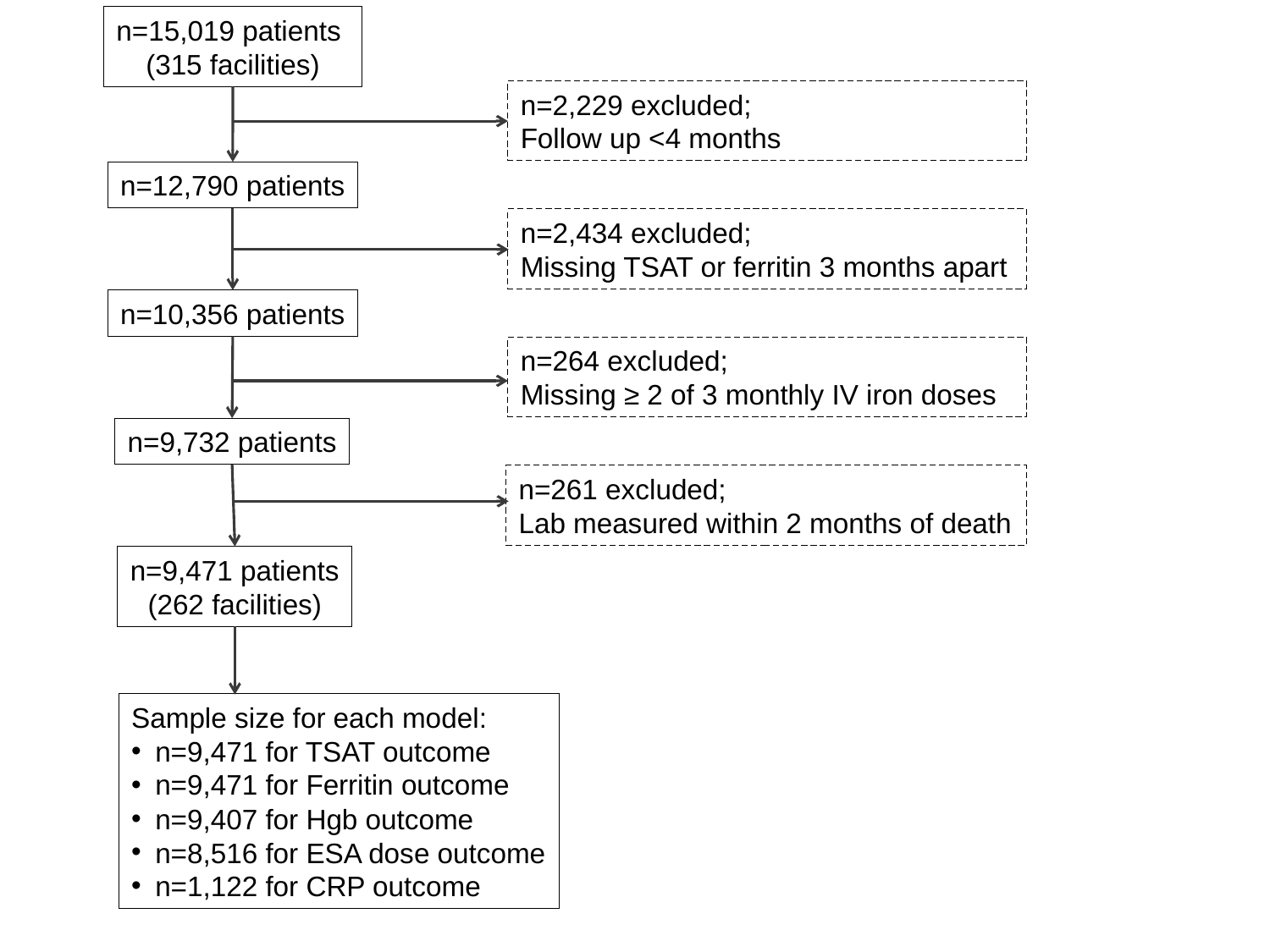

n=15,019 patients
(315 facilities)
n=2,229 excluded;
Follow up <4 months
n=12,790 patients
n=2,434 excluded;
Missing TSAT or ferritin 3 months apart
n=10,356 patients
n=264 excluded;
Missing ≥ 2 of 3 monthly IV iron doses
n=9,732 patients
n=261 excluded;
Lab measured within 2 months of death
n=9,471 patients
(262 facilities)
Sample size for each model:
n=9,471 for TSAT outcome
n=9,471 for Ferritin outcome
n=9,407 for Hgb outcome
n=8,516 for ESA dose outcome
n=1,122 for CRP outcome
