# Supplementary material for: Evaluating the effectiveness of IV iron dosing for anemia management in common clinical practice: results from the Dialysis Outcomes and Practice Patterns Study (DOPPS)
Source: BMC Nephrol. 2017 Nov 9;18:330. doi: 10.1186/s12882-017-0745-9 (PMC5679150; doi:10.1186/s12882-017-0745-9)
Supplement: Supplementary file 2 — Schematic of timing of IV iron dose, ESA dose, and laboratory measures in analytic models. (PPTX 529 kb) [file 12882_2017_745_MOESM2_ESM.pptx]

## Slide 1
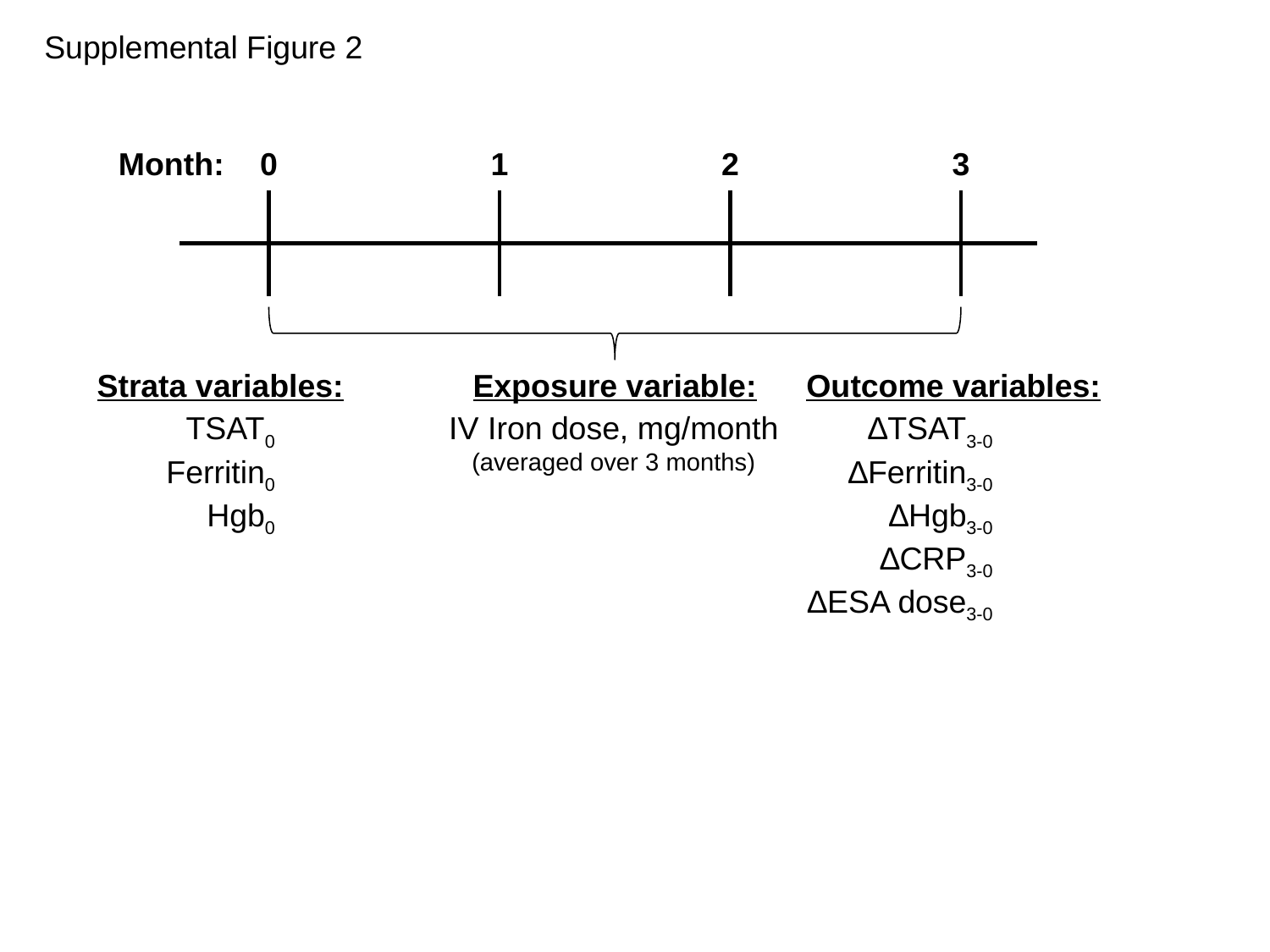

Supplemental Figure 2
Month:
0
1
2
3
Strata variables:
TSAT0
Ferritin0
Hgb0
Exposure variable:
IV Iron dose, mg/month
(averaged over 3 months)
Outcome variables:
∆TSAT3-0
∆Ferritin3-0
∆Hgb3-0
∆CRP3-0
∆ESA dose3-0
