# Supplementary material for: Evaluating the effectiveness of IV iron dosing for anemia management in common clinical practice: results from the Dialysis Outcomes and Practice Patterns Study (DOPPS)
Source: BMC Nephrol. 2017 Nov 9;18:330. doi: 10.1186/s12882-017-0745-9 (PMC5679150; doi:10.1186/s12882-017-0745-9)
Supplement: Supplementary file 3 — Distribution of 3-month IV iron dose. (PPTX 546 kb) [file 12882_2017_745_MOESM3_ESM.pptx]

## Slide 1
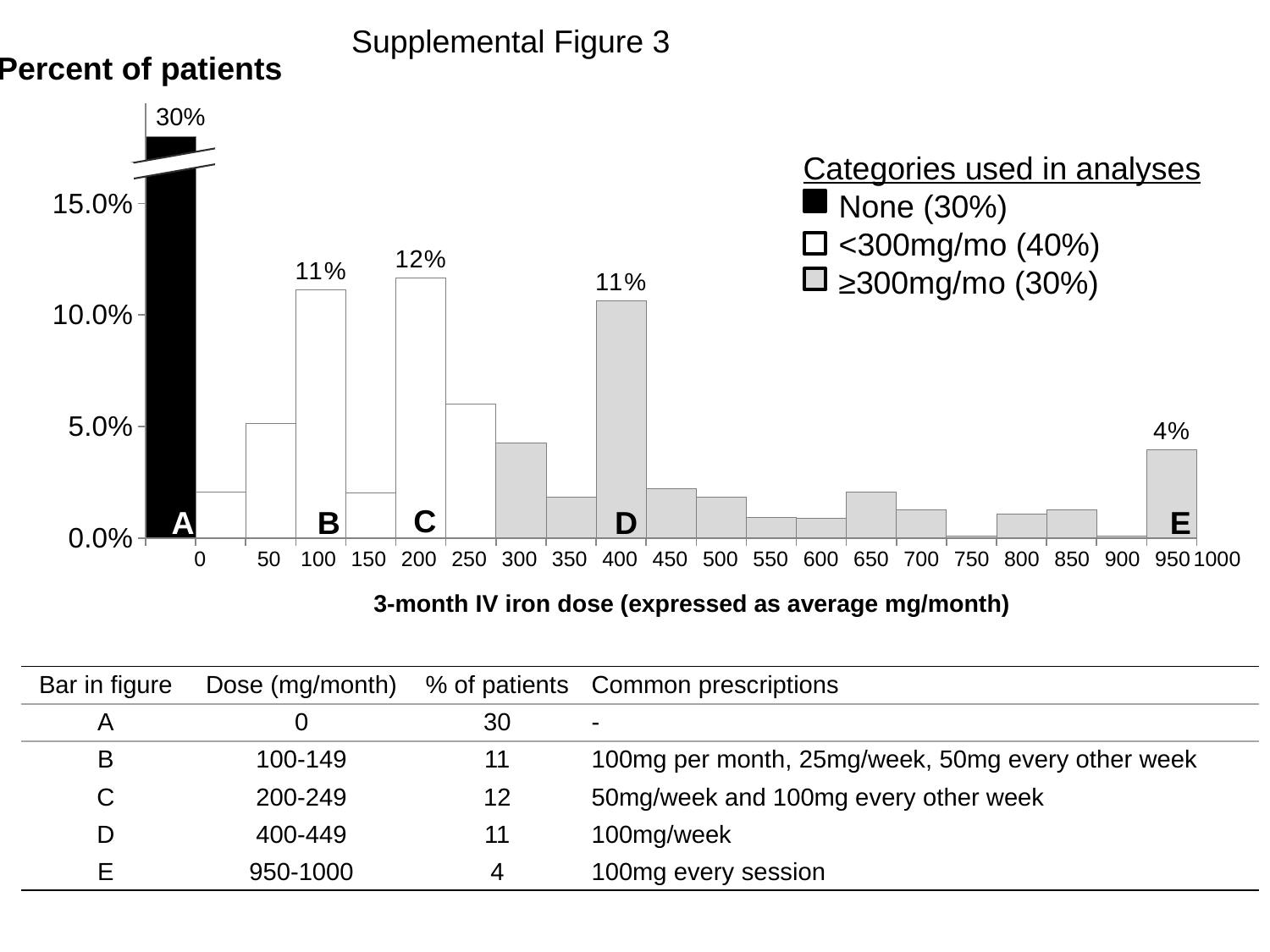

# 3mo IV iron dose distribution
Supplemental Figure 3
### Chart
| Category | % with 0 |
|---|---|
| 0 | 0.18 |
| 50 | 0.020800337873508604 |
| 100 | 0.05120895364797804 |
| 150 | 0.11139267236828213 |
| 200 | 0.020166825044873826 |
| 250 | 0.1165663604687995 |
| 300 | 0.060078133248864955 |
| 350 | 0.04244535951853025 |
| 400 | 0.018477457501847747 |
| 450 | 0.10643015521064302 |
| 500 | 0.022067363530778164 |
| 550 | 0.018371872030408617 |
| 600 | 0.009397106958082569 |
| 650 | 0.008974765072326049 |
| 700 | 0.020483581459191217 |
| 750 | 0.012670256572695597 |
| 800 | 0.0010558547143912998 |
| 850 | 0.010769718086791258 |
| 900 | 0.012670256572695597 |
| 950 | 0.0007390983000739098 |
| 1000 | 0.03959455178967374 |Percent of patients
30%
Categories used in analyses
 None (30%)
 <300mg/mo (40%)
 ≥300mg/mo (30%)
C
A
B
D
E
| 0 | 50 | 100 | 150 | 200 | 250 | 300 | 350 | 400 | 450 | 500 | 550 | 600 | 650 | 700 | 750 | 800 | 850 | 900 | 950 | 1000 |
| --- | --- | --- | --- | --- | --- | --- | --- | --- | --- | --- | --- | --- | --- | --- | --- | --- | --- | --- | --- | --- |
3-month IV iron dose (expressed as average mg/month)
| Bar in figure | Dose (mg/month) | % of patients | Common prescriptions |
| --- | --- | --- | --- |
| A | 0 | 30 | - |
| B | 100-149 | 11 | 100mg per month, 25mg/week, 50mg every other week |
| C | 200-249 | 12 | 50mg/week and 100mg every other week |
| D | 400-449 | 11 | 100mg/week |
| E | 950-1000 | 4 | 100mg every session |
