# Supplementary material for: Evaluating the effectiveness of IV iron dosing for anemia management in common clinical practice: results from the Dialysis Outcomes and Practice Patterns Study (DOPPS)
Source: BMC Nephrol. 2017 Nov 9;18:330. doi: 10.1186/s12882-017-0745-9 (PMC5679150; doi:10.1186/s12882-017-0745-9)
Supplement: Supplementary file 4 — Joint distributions of 3-month and 12-month IV iron doses (% of patients). (DOCX 34 kb) [file 12882_2017_745_MOESM4_ESM.docx]

### Table S1: Joint distributions of 3-month and 12-month IV iron doses (% of patients)

|  | | | **12-month IV iron dose (mg/mo)** | | | | | |  | |
| --- | --- | --- | --- | --- | --- | --- | --- | --- | --- | --- |
|  |  |  | **0** | | **<300** | | **≥300** | | **Total (row)** | |
| **3-month IV iron dose (mg/mo)** | | **0** | 11% | | 17% | | 1% | | 29% | |
|  |  | **<300** | 0% | | 36% | | 5% | | 41% | |
|  |  | **≥300** | 0% | | 11% | | 19% | | 30% | |
|  | | **Total (column)** | | 11% | 63% | | 26% | | 100% | |

Footnote: n=6,155. This is a subset of the sampled patients who had iron use information for the first 12mo after study entry. This excluded n=3,316 (35% of the analysis sample) who did not have 12 months of IV iron use reported. Of patients with 12-month data, 66% remained in the same IV iron dose category (diagonal) from the 3-month to 12-month period.

###

### Figure S1: Flow Chart for Selection of Analysis Sample

**Figure S2: Schematic of timing of IV iron dose, ESA dose, and laboratory measures in analytic models**

**Figure S3: Distribution of 3-month IV iron dose**

Each stratum represents a separate model (14 total per figure) adjusted for age, sex, black race, time on dialysis, catheter use, BMI, region (Europe-ANZ, North America), 13 comorbid conditions, and the following measures at month 0: Hgb, white blood cell count; serum albumin, creatinine, ferritin, TSAT; and 3-month ESA dose (unless included in the outcome or strata). The vertical bars indicate 95% confidence intervals.

**Figure S4: Adjusted change in hemoglobin, TSAT, or ferritin from before to after IV iron dosing, dividing ≥300 mg/month into** **maintenance and replacement dosing categories.** Replacement dosing defined as ≥500 mg during any of the 3 months, and maintenance dosing defined as 300-499 mg per month in all 3 months. Each stratum represents a separate model (14 total per figure) adjusted for age, sex, black race, time on dialysis, catheter use, BMI, region (Europe-ANZ, North America), 13 comorbid conditions, and the following measures at month 0: Hgb, white blood cell count; serum albumin, creatinine, ferritin, TSAT; and 3-month ESA dose (unless included in the outcome or strata). The vertical bars indicate 95% confidence intervals.

**Figure S5: Adjusted change in Hemoglobin, from before to after IV iron dosing stratified by ESA Dose and TSAT**

Each stratum represents a separate model (20 total) adjusted for age, sex, black race, time on dialysis, catheter use, BMI, region (Europe-ANZ, North America), 13 comorbid conditions, and the following measures at month 0: white blood cell count; serum albumin, creatinine, ferritin. The vertical bars indicate 95% confidence intervals.

**Figure S6: Adjusted change in 1-month Hemoglobin, TSAT, or Ferritin from before to after IV iron dosing**

Each stratum represents a separate model (14 total per figure) adjusted for age, sex, black race, time on dialysis, catheter use, BMI, region (Europe-ANZ, North America), 13 comorbid conditions, and the following measures at month 0: Hgb, white blood cell count; serum albumin, creatinine, ferritin, TSAT; and 1-month ESA dose (unless included in the outcome or strata). The vertical bars indicate 95% confidence intervals.

**Figure S7: Adjusted change in CRP, from before to after IV iron dosing**

Each stratum represents a separate model (14 total per figure) adjusted for age, sex, black race, time on dialysis, catheter use, BMI, region (Europe-ANZ, North America), 13 comorbid conditions, and the following measures at month 0: Hgb, white blood cell count; serum albumin, creatinine, ferritin, TSAT; and 3-month ESA dose (unless included in the outcome or strata). The vertical bars indicate 95% confidence intervals.
