# Supplementary material for: Evaluating the effectiveness of IV iron dosing for anemia management in common clinical practice: results from the Dialysis Outcomes and Practice Patterns Study (DOPPS)
Source: BMC Nephrol. 2017 Nov 9;18:330. doi: 10.1186/s12882-017-0745-9 (PMC5679150; doi:10.1186/s12882-017-0745-9)
Supplement: Supplementary file 5 — Adjusted change in hemoglobin, TSAT, or ferritin from before to after IV iron dosing, dividing ≥ 300 mg/month into maintenance and replacement dosing categories. (PPTX 89 kb) [file 12882_2017_745_MOESM5_ESM.pptx]

## Slide 1
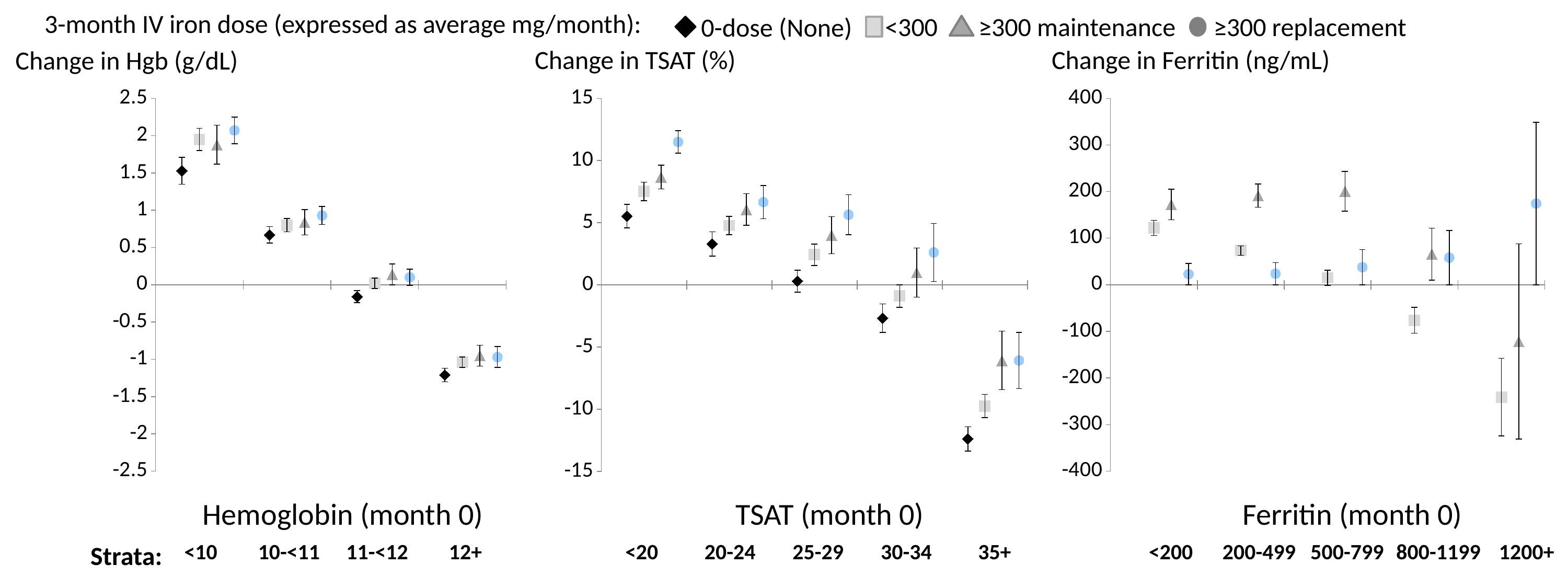

3-month IV iron dose (expressed as average mg/month):
<300
≥300 maintenance
≥300 replacement
0-dose (None)
Change in TSAT (%)
Change in Ferritin (ng/mL)
Change in Hgb (g/dL)
### Chart
| Category | 0-dose (None) | <300 | ≥300 no Bolus | ≥300 Bolus |
|---|---|---|---|---|
### Chart
| Category | 0-dose (None) | <300 | ≥300 no bolus | ≥300 Bolus |
|---|---|---|---|---|
### Chart
| Category | 0-dose (None) | <300 | ≥300 no bolus | ≥300 Bolus |
|---|---|---|---|---|Hemoglobin (month 0)
TSAT (month 0)
Ferritin (month 0)
Strata:
| <10 | 10-<11 | 11-<12 | 12+ | | <20 | 20-24 | 25-29 | 30-34 | 35+ | | <200 | 200-499 | 500-799 | 800-1199 | 1200+ |
| --- | --- | --- | --- | --- | --- | --- | --- | --- | --- | --- | --- | --- | --- | --- | --- |
