# Supplementary material for: Evaluating the effectiveness of IV iron dosing for anemia management in common clinical practice: results from the Dialysis Outcomes and Practice Patterns Study (DOPPS)
Source: BMC Nephrol. 2017 Nov 9;18:330. doi: 10.1186/s12882-017-0745-9 (PMC5679150; doi:10.1186/s12882-017-0745-9)
Supplement: Supplementary file 6 — Adjusted change in Hemoglobin, from before to after IV iron dosing stratified by ESA Dose and TSAT. (PPTX 117 kb) [file 12882_2017_745_MOESM6_ESM.pptx]

## Slide 1
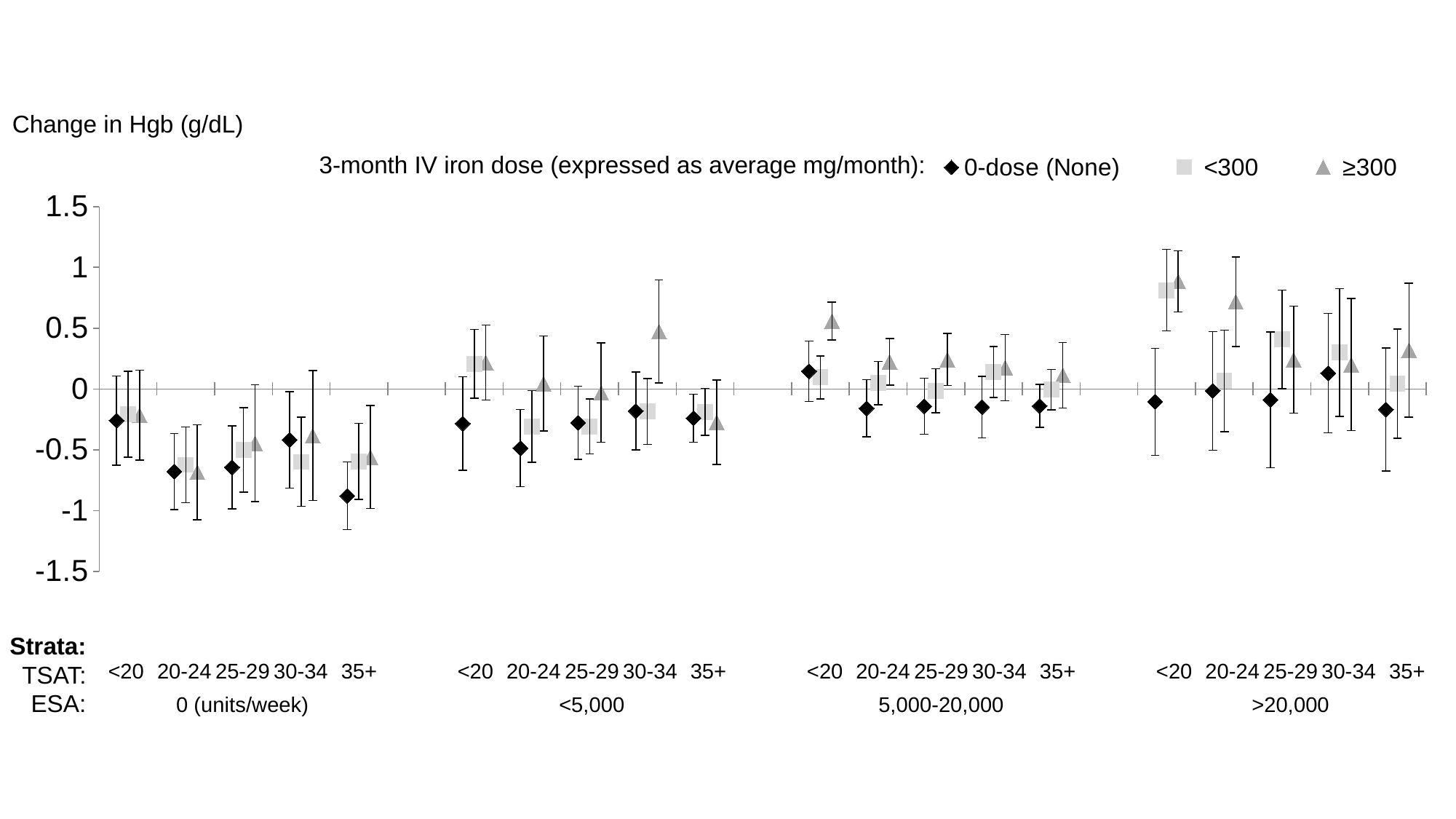

Change in Hgb (g/dL)
### Chart
| Category | 0-dose (None) | <300 | ≥300 |
|---|---|---|---|3-month IV iron dose (expressed as average mg/month):
Strata:
TSAT:
ESA:
| <20 | 20-24 | 25-29 | 30-34 | 35+ | | <20 | 20-24 | 25-29 | 30-34 | 35+ | | <20 | 20-24 | 25-29 | 30-34 | 35+ | | <20 | 20-24 | 25-29 | 30-34 | 35+ |
| --- | --- | --- | --- | --- | --- | --- | --- | --- | --- | --- | --- | --- | --- | --- | --- | --- | --- | --- | --- | --- | --- | --- |
| 0 (units/week) | | | | | | <5,000 | | | | | | 5,000-20,000 | | | | | | >20,000 | | | | |
