# Supplementary material for: Evaluating the effectiveness of IV iron dosing for anemia management in common clinical practice: results from the Dialysis Outcomes and Practice Patterns Study (DOPPS)
Source: BMC Nephrol. 2017 Nov 9;18:330. doi: 10.1186/s12882-017-0745-9 (PMC5679150; doi:10.1186/s12882-017-0745-9)
Supplement: Supplementary file 8 — Adjusted change in CRP, from before to after IV iron dosing. (PPTX 56 kb) [file 12882_2017_745_MOESM8_ESM.pptx]

## Slide 1
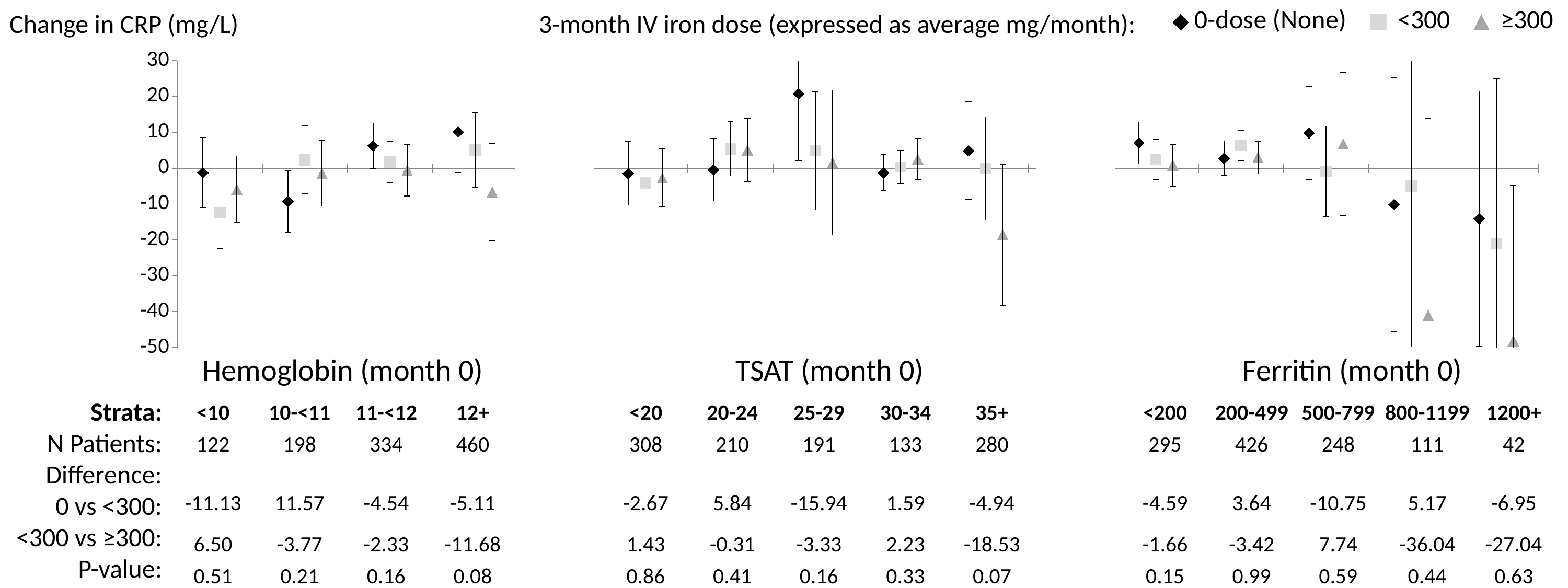

### Chart
| Category | 0-dose (None) | <300 | ≥300 |
|---|---|---|---|Change in CRP (mg/L)
3-month IV iron dose (expressed as average mg/month):
Hemoglobin (month 0)
TSAT (month 0)
Ferritin (month 0)
Strata:
N Patients:
Difference:
0 vs <300:
<300 vs ≥300:
P-value:
| <10 | 10-<11 | 11-<12 | 12+ | | <20 | 20-24 | 25-29 | 30-34 | 35+ | | <200 | 200-499 | 500-799 | 800-1199 | 1200+ |
| --- | --- | --- | --- | --- | --- | --- | --- | --- | --- | --- | --- | --- | --- | --- | --- |
| 122 | 198 | 334 | 460 | | 308 | 210 | 191 | 133 | 280 | | 295 | 426 | 248 | 111 | 42 |
| | | | | | | | | | | | | | | | |
| -11.13 | 11.57 | -4.54 | -5.11 | | -2.67 | 5.84 | -15.94 | 1.59 | -4.94 | | -4.59 | 3.64 | -10.75 | 5.17 | -6.95 |
| 6.50 | -3.77 | -2.33 | -11.68 | | 1.43 | -0.31 | -3.33 | 2.23 | -18.53 | | -1.66 | -3.42 | 7.74 | -36.04 | -27.04 |
| 0.51 | 0.21 | 0.16 | 0.08 | | 0.86 | 0.41 | 0.16 | 0.33 | 0.07 | | 0.15 | 0.99 | 0.59 | 0.44 | 0.63 |
